# Identification of a cellular senescence-related-lncRNA (SRlncRNA) signature to predict the overall survival of glioma patients and the tumor immune microenvironment

**DOI:** 10.3389/fgene.2023.1096792

**Published:** 2023-02-24

**Authors:** Qing Liu, Hongbo Bao, Sibin Zhang, Tianjun Song, Chenlong Li, Guiyin Sun, Xiaoyang Sun, Tianjiao Fu, Yujie Wang, Peng Liang

**Affiliations:** ^1^ Department of Neurosurgery, Harbin Medical University Cancer Hospital, Harbin, China; ^2^ Department of Medicine II, University Hospital LMU Munich, Munich, Germany

**Keywords:** cellular senescence, glioma, long non-coding RNA, prognostic signature, overall survival, immune microenvironment

## Abstract

**Background:** Gliomas are brain tumors that arise from glial cells, and they are the most common primary intracranial tumors with a poor prognosis. Cellular senescence plays a critical role in cancer, especially in glioma. In this study, we constructed a senescence-related lncRNA (SRlncRNA) signature to assess the prognosis of glioma.

**Methods:** The Cancer Genome Atlas was used to collect SRlncRNA transcriptome profiles and clinical data about glioma. Patients were randomized to training, testing, and whole cohorts. LASSO and Cox regression analyses were employed to construct the SRlncRNA signature, and Kaplan–Meier (K-M) analysis was performed to determine each cohort’s survival. Receiver operating characteristic (ROC) curves were applied to verify the accuracy of this signature. Gene set enrichment analysis was used to visualize functional enrichment (GSEA). The CIBERSORT algorithm, ESTIMATE and TIMER databases were utilized to evaluate the differences in the infiltration of 22 types of immune cells and their association with the signature. RT–qPCR and IHC were used to identify the consistency of the signature in tumor tissue.

**Results:** An SRlncRNA signature consisting of six long non-coding RNAs (lncRNAs) was constructed, and patients were divided into high-risk and low-risk groups by the median of their riskscore. The KM analysis showed that the high-risk group had worse overall survival, and the ROC curve confirmed that the riskscore had more accurate predictive power. A multivariate Cox analysis and its scatter plot with clinical characteristics confirmed the riskscore as an independent risk factor for overall survival. GSEA showed that the GO and KEGG pathways were mainly enriched in the immune response to tumor cells, p53 signaling pathway, mTOR signaling pathway, and Wnt signaling pathway. Further validation also yielded significant differences in the risk signature in terms of immune cell infiltration, which may be closely related to prognostic differences, and qRT–PCR and IHC confirmed the consistency of the expression differences in the major lncRNAs with those in the prediction model.

**Conclusion** Our findings indicated that the SRlncRNA signature might be used as a predictive biomarker and that there is a link between it and immune infiltration. This discovery is consistent with the present categorization system and may open new avenues for research and personalized therapy.

## 1 Introduction

Glioma, the most common malignant tumor of the central nervous system (CNS), accounts for approximately 75% of all malignancies and 30%–40% of all CNS primary tumors, with a median overall survival of 9–12 months (Lapointe et al., 2018; Chen et al., 2020). According to WHO CNS5 in 2021, the importance of grading within tumor type was proclaimed (Louis et al., 2021). Four different families are divided: 1) Adult-type diffuse gliomas; 2) Pediatric-type diffuse low-grade gliomas; 3) Pediatric-type diffuse high-grade gliomas; and 4) Circumscribed astrocytic gliomas. On the other hand, adult-type diffuse gliomas include only three types: Astrocytoma, IDH-mutant; Oligodendroglioma, IDH-mutant and 1p/19q-codeleted; and Glioblastoma, IDH-wildtype; The latest edition of WHO Classification of Central Nervous System Neoplasm will be the first of its kind Genotypes were included in the diagnosis of glioma, in which isocitrate dehydrogenase was included Genes (isocitrate dehydrogenase, IDH) and chromosomes 1p/19q is the core basis of glioma molecular typing, according to IDH mutation Histologically similar diffuse gliomas are divided into different subgroups Type I, while the 1p/19q joint deletion in IDH mutant oligodendrocyte fine The significance of neoplasms is gaining recognition, and this new typing method can Better judgment of prognosis, accurate guidance of treatment (Brandner et al., 2022; Xu et al., 2022). Despite advances in different therapies, such as surgery, radiation, and chemotherapy, in many instances, these glioma patients’ overall survival rates have declined. Glioblastoma patients, in particular, have a 14-month average survival (Delgado-Lopez and Corrales-Garcia, 2016). The extensive infiltration of cancer cells into the brain tissue complicates full surgical resection of glioma and is a fundamental factor in glioma’s poor prognosis (Verburg et al., 2016). Glioma cells, in contrast to normal tissue, exhibit an uninhibited pattern of tumor growth, including the maintenance of proliferative activity, resistance to growth inhibitory factors and cell death stimuli, stimulation of angiogenesis, promotion of invasion and metastasis, reprogramming of energy metabolism, and immune escape (Hainaut and Plymoth, 2013). Glioma displays not only these properties but also a dramatically heterogeneous tumorigenic “tumor microenvironment” by attracting undifferentiated and differentiated cells to generate glioma cell heterogeneity, boosting their resistance to chemotherapy and radiation (Saadatpour et al., 2016; Quezada et al., 2018). Therefore, there is an urgent need to better understand the mechanisms and development of glioma at the genetic and molecular levels, which in turn will provide a new theoretical basis for the diagnosis and treatment of gliomas.

Cellular senescence was first discovered in 1961 by microbiologists Leonard Hayflick and Paul Moorhead during *in vitro* passaging of adult cells. Cellular senescence is a self-protective mechanism that occurs in response to DNA damage, telomere shortening, oncogene activation, epigenetic changes, and oxidative stress, manifesting itself as permanent growth arrest and cell division cycle arrest in G1 or S phase to prevent the transmission of abnormal genes to the next-generation of cells, thereby maintaining homeostasis (Munoz-Espin and Serrano, 2014; Hernandez-Segura et al., 2018; Calcinotto et al., 2019; Mehdizadeh et al., 2022). Depending on the cause of senescence, cell senescence can be categorized into replicative senescence and stress-induced premature senescence, the latter of which can be subdivided into DNA-damaged senescence, oxidative stress senescence, epigenetic senescence, and inflammatory senescence (Hernandez-Segura et al., 2018; Mehdizadeh et al., 2022). Cell senescence is closely associated with tumorigenesis, progression, and resistance to therapy. Oncogene-induced senescence (OIS) is a term for senescence induced by oncogene activation. Endothelial cellular senescence leading to the secretion of CXCL11 increases the aggressiveness of breast cancer cells (Hwang et al., 2020). Cellular senescence promotes skin carcinogenesis through p38MAPK and p44/42 MAPK signaling (Alimirah et al., 2020). Reversible cellular senescence is involved in the response of colon cancer to methotrexate (Dabrowska et al., 2021). Thus, senescence is one of the physiological inhibitory pathways that suppresses tumor lesions. On the other hand, inactivation of oncogenes can induce senescence. Inactivation of MYC leads to cell senescence and degeneration in a variety of tumor types, including lymphoma, osteosarcoma, and hepatocellular carcinoma. These effects are mediated by multiple mechanisms, reflecting the complexity of oncogene-induced tumorigenesis and cell senescence (Bellovin et al., 2013).

Non-coding RNAs include microRNAs (miRNAs, 18–22 nt) and long non-coding RNAs (longer than 200 nt) (Peng et al., 2018; Cheng et al., 2020; Gao et al., 2020; Garbo et al., 2022). LncRNAs were originally identified by large-scale sequencing of a full-length mouse cDNA library (Okazaki et al., 2002). LncRNAs can be found in the nucleus or cytoplasm and play a variety of roles depending on their subcellular location. In the nucleus, lncRNAs might have a role in gene expression regulation, transcriptional control, and mRNA splicing. They can alter mRNA stability and protein function in the cytoplasm (Quinn and Chang, 2016). Furthermore, lncRNAs perform their roles through a variety of molecular processes, including DNA binding to regulate gene transcription, binding to proteins, producing short functional peptides, and regulating the posttranscriptional stages by acting as competitive endogenous RNAs (ceRNAs) or miRNA sponges (Kopp and Mendell, 2018; Chen et al., 2021; Mao et al., 2022). Although lncRNAs are known to play roles in a variety of human diseases, the mechanism by which lncRNAs drive glioma cell senescence is unexplored; hence, we screened the TCGA database for cell senescence-associated lncRNAs to develop a novel algorithm to predict the outcome of glioma.

## 2 Methods

### 2.1 Data source

The clinical and gene expression data of patients with LGGGBM were obtained from the TCGA database (https://cancergenome.nih.gov/) and the CGGA database (http://www.cgga.org.cn/). Normal brain RNA sequencing data were obtained from GTEx and utilized as normal control data. In this research, data from 609 glioma samples and 1152 normal brain samples were analyzed. Among them, the data used in the gene expression data were normalized. Next, DEGs were chosen using the R software’s “limma” package with the absolute value of the log2-transformed fold change (FC) > 1 and the adjusted *p*-value (adj. P) < 0.05 as the cutoff. In addition, the ComBat method was used to reduce batch effects with the R package “sva”.

### 2.2 Screening of lncRNAs and cellular senescence-related genes

The profiles of lncRNAs were acquired from the RNAseq dataset. The log2 transformation was used to normalize the total RNA expression data. The list of cellular senescence-related genes was obtained from the Human Aging Genomic Resources (HAGR) (https://genomics.senescence.info). The Pearson correlation was used to calculate the relationship between lncRNAs and cellular senescence-related genes. Cellular senescence-related lncRNAs (SRlncRNAs) were screened by a correlation coefficient *R* ^2^ > 0.4 and a *p* < 0.001. Finally, coexpression networks were visualized using Cytoscape software 3.8.0.

### 2.3 Identification of prognostic cellular senescence-related lncRNAs

To construct a validated prognostic model, 609 glioma patients were randomly divided into training and testing cohorts. Ultimately, 306 patients were enrolled in the training cohort, and 303 patients were enrolled in the testing cohort. The key characteristics of each cohort are shown in Table 1. The SRlncRNA signature was derived based on the training cohort, and its potential to predict patient survival was validated utilizing the testing cohort and the whole cohort. We also confirmed the prognostic signature in the TCGA-LGG cohort, the TCGA-GBM cohort and the CGGA cohort (Supplementary Tables S1, S2).

**TABLE 1:** The characteristics of glioma patients from TCGA in this study.

Variable	Group	Overall cohort (*n* = 609)	Training cohort (*n* = 306)	Testing cohort (*n* = 303)
Age	≤65	528 (87.0%)	268 (87.6%)	260 (85.8%)
	>65	81 (13.0%)	38 (12.4%)	43 (14.2%)
Gender	Female	262 (43.0%)	132 (43.1%)	130 (42.9%)
	Male	347 (57.0%)	174 (56.9%)	173 (57.1%)
Tumor grade	G2	229 (37.6%)	116 (37.9%)	113 (37.3%)
	G3	244 (40.0%)	130 (42.5%)	114 (37.6%)
	G4	136 (22.4%)	60 (19.6%)	76 (25.1%)
IDH mutant status	Mutant	397 (65.2%)	202 (66.0%)	195 (64.4%)
	Wildtype	212 (34.8%)	104 (34.0%)	108 (35.6%)
1p19q codeletion status	Non-codel	453 (74.4%)	227 (74.2%)	226 (74.6%)
	Codel	156 (25.6%)	79 (25.8%)	77 (25.4%)
Survival status	Alive	414 (68.0%)	212 (69.3%)	202 (66.7%)
	Dead	195 (32.0%)	94 (30.7%)	101 (33.3%)
Survivaltime (years) (Mean ± SD)		2.16 ± 2.35	2.17 ± 2.33	2.15 ± 2.36

The prognostic significance of cellular senescence-related lncRNAs was initially determined using univariate Cox regression. Least absolute shrinkage and selection operator (LASSO) regression was used to integrate the cellular senescence-related lncRNAs with *p* < 0.05 in univariate analysis. The LASSO results were then included in a multivariate Cox model to generate a risk score. A risk score was calculated using a linear combination of cellular senescence-related lncRNA expression levels multiplied by a regression coefficient (β): risk score = $\sum_{i=1}^{n}\beta_{i}\times$ (expression of lncRNA_i_). Based on the median risk score, the patients were categorized into high-risk and low-risk groups. The log-rank test was used to compare the survival differences between the two groups.

### 2.4 Prognostic model construction and validation

To construct an independent prognostic model, Cox regression was used. Patient survival was predicted utilizing a nomogram. The correctness of the model was assessed by utilizing the concordance index (C-index), calibration curves, and receiver operating characteristic (ROC) curves. We used multivariate Cox regression with demographic data to determine whether the risk score was an independent predictor of patient outcomes.

### 2.5 Functional analysis

The functional enrichment of the gene expression data was interpreted by employing Gene Set Enrichment Analysis (GSEA, http://www.broadinstitute.org/gsea/index.jsp). The Gene Ontology (GO) and Kyoto Encyclopedia of Genes and Genomes (KEGG) pathways associated with cellular senescence are displayed, as well as the functional enrichment of cell senescence-related lncRNAs with predictive significance.

### 2.6 Correlation analysis of immune cell infiltration

To assess the riskscore and the level of correlation of the immune Microenvironment, immune-infiltrating scores of 16 immune cells, 13 immune function and immune checkpoint were calculated by singlesample gene set enrichment analysis (ssGSEA) using the “gsva” and “GSEABase” packages in R software. Additionally examined was the relationship between riskscore and immunological subtypes (C1–C6). At the same time, we calculated the immune infiltration between the high- and low-risk groups using the CIBERSORT, TIMER and ESTIMATE databases. Pearson correlation was used to determine the relationship between risk scores and immune infiltration.

### 2.7 Clinical samples and cell culture

All procedures for this research were approved by the Ethics Committee of Harbin Medical University (Harbin, China). Glioma samples and adjacent normal brain tissue were collected from 24 patients who were resected at Harbin Medical University Cancer Hospital. Patients had not been treated with radiotherapy or chemotherapy before surgery. All tissue samples were pathologically confirmed and immediately frozen in liquid nitrogen for storage until the RNA was extracted. Each patient signed a written informed consent form before the tissue samples were used for research purposes. The clinical characteristics of the 24 glioma patients are shown in Supplementary Table S4.

The human GBM cell lines (T98G and U251) and the normal human astrocyte cell line (NHA) were obtained from our laboratory (Li et al., 2019a). All cell lines were cultured in Dulbecco’s modified Eagle’s medium (DMEM; SIGMA) supplemented with 10% fetal bovine serum (FBS; PAN SERATECH) at 37°C in a humidified chamber containing 5% CO_2_. All glioma patients from our hospital were informed and ethical approval for the current research was obtained through the Ethics Committee of Harbin Medical University (KY2021-42).

### 2.8 qRT–PCR analysis

The RNAsimple Total RNA Kit (DP419, Tiangen Biotech Co., Ltd. Beijing, China) was used to extract total RNA from tissue and cells. The lnRcute lncRNA First-Strand cDNA Kit (KR202, Tiangen Biotech Co., Ltd.) was used to construct complementary DNA. The lnRcute lncRNA qPCR Kit (FP402, Tiangen Biotech Co., Ltd.) was used to perform RT-qPCRs according to the manufacturer’s instructions. To evaluate relative gene expression, the 2^−ΔΔCt^ method was utilized. GAPDH was used as an endogenous control. The primer sequences were as follows: SNAI3-AS1, 5-TGA​GGT​GCT​CCT​CCG​AGA​AT-3 (Forward) and 5-GTA​TAG​CTC​CCT​GGC​AGA​GTT​CA-3 (Reverse); GAPDH, 5-CAG​GAG​GCA​TTG​CTG​ATG​AT-3 (Forward) and 5GAA​GGC​TGG​GGC​TCA​TTT-3 (Reverse).

### 2.9 Immunohistochemistry

All tissues were fixed overnight in formalin solution, dehydrated in ethanol, embedded in paraffin and sectioned at 5 mm. To remove the paraffin, specimens were treated with xylene and ethanol. Slides were blocked with 5% normal goat serum and incubated overnight at 4°C with anti-YPEL3 (Proteintech). After washing with PBS, slides were incubated with goat anti-rabbit horseradish peroxidase for 30 min at room temperature. Immunohistochemical (IHC) reactions were detected using the DAB kit. Slides were examined under a phase-contrast light microscope (Nikon).

### 2.10 Statistical analysis

The survival curves were constructed using the Kaplan-Meier method and evaluated using the log-rank test. The predictive impact of the cellular SRlncRNAS signature and clinicopathological data was estimated by combining Cox regression and LASSO regression. The statistical analyses were carried out using the R programming language (version 4.1.3). The differences between groups were calculated utilizing a two-tailed Student’s *t*-test. The statistical tests were conducted bilaterally, with a significance level of *p* < 0.05.

## 3 Results

### 3.1 Cellular senescence-related lncRNAs with significant prognostic value in glioma

**FIGURE 1:**
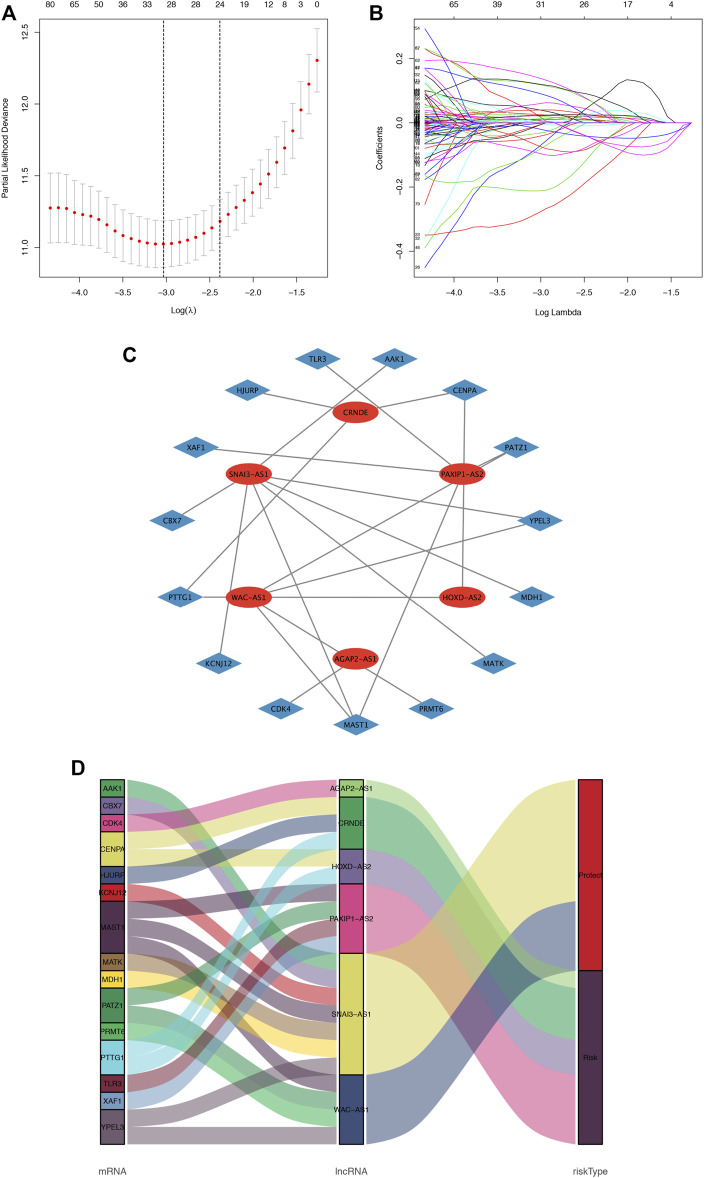
Cellular senescence-related lncRNA selection utilizing the LASSO model and the coexpression network and Sankey diagram. **(A)**. LASSO coefficient values and vertical dashed lines generated at the best log (lambda) values, **(B)**. LASSO coefficient curves for SRlncRNAs. **(C)**. The coexpression network between SRlncRNAs and mRNAs in glioma. The red nodes represent prognostic lncRNAs, and the sky-blue nodes represent mRNAs. **(D)** Sankey diagram showing the association between prognostic cell senescence-related lncRNAs, mRNAs, and risk types.

The flowchart of this study is shown in Supplementary Figure S1. A total of 279 cellular senescence-related genes were obtained from HAGR, among which 273 genes were expressed in glioma, and a total of 483 cellular SRlncRNAs were obtained by constructing a cellular senescence-related mRNA and lncRNA coexpression network. Applying LASSO regression, 31 lncRNAs associated with cell senescence were identified (Figures 1A, B; Supplementary Table S3). In total, 13 lncRNAs with a low risk (hazard ratio (HR) < 1) and 18 lncRNAs with a high risk (hazard ratio (HR) > 1) were found. Furthermore, multivariate Cox analysis revealed 6 lncRNAs with prognostic significance and their coefficients among the aforementioned 31 cellular SRlncRNAs (Table 2). The names of the 6 lncRNAs were SNAI3-AS1, CRNDE, AGAP2-AS1, HOXD-AS2, PAXIP1-AS2, WAC-AS1. These 6 lncRNAs were exploited to construct an SRlncRNA signature. The risk score was calculated using the following formula: (0.54993 × CRNDE) + (0.11751 × AGAP2-AS1) + (0.28073 × HOXD-AS2) + (0.46398 × PAXIP1-AS2)—(0.45368 × WAC-AS1)- (1.23692 × SNAI3-AS1). According to the hazard ratio (HR) score obtained by the multivariate Cox regression analysis, CRNDE, AGAP2-AS1, HOXD-AS2, and PAXIP1-AS2 were risk factors, whereas SNAI3-AS1 and WAC-AS1 were protective factors. These six lncRNAs were used to construct an optimal prognostic risk model and a prognostic visual coexpression network of cellular SRlncRNAs-mRNAs (Figures 1C, D).

**TABLE 2:** The 6 senescence-related prognostic lncRNA multivariate Cox regression analyses of OS in glioma patients.

LncRNA	Coefficient	HR	HR.95 L	HR.95 H	*p*-Value
SNAI3-AS1	−1.23692	0.29028	0.11743	0.71753	0.00739
CRNDE	0.54993	1.73312	1.26485	2.37476	0.00062
AGAP2-AS1	0.11751	1.12469	0.96507	1.31070	0.13239
HOXD-AS2	0.28073	1.32409	0.90819	1.93046	0.14447
PAXIP1-AS2	0.46398	1.59040	1.08875	2.32318	0.01641
WAC-AS1	−0.45368	0.63529	0.39669	1.01740	0.05900

**FIGURE 2:**
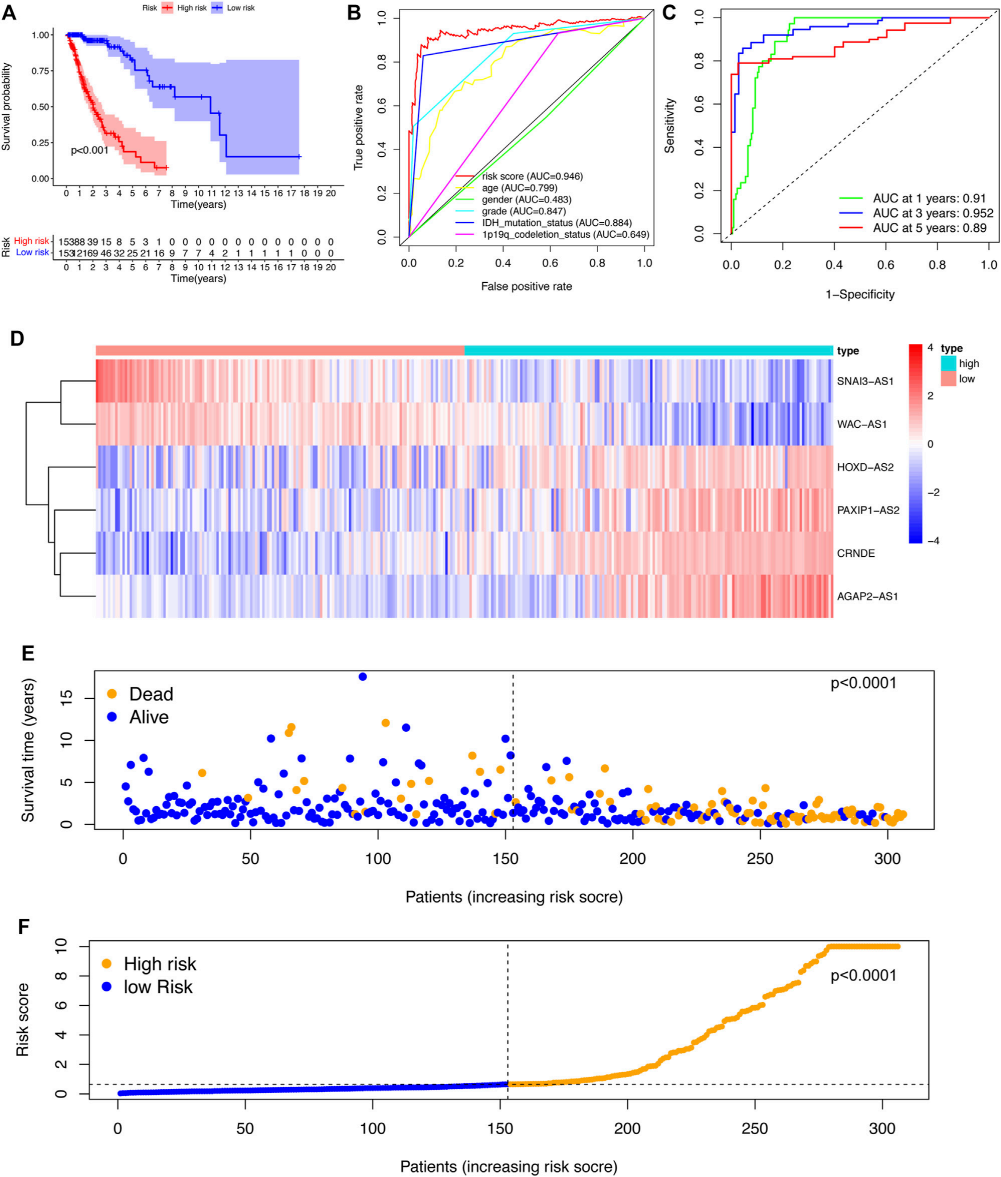
Construction and evaluation of the SRlncRNA signature in the training cohort. **(A)** K-M curves indicated that the high-risk group had a lower survival rate. **(B)** ROC curves and AUC values for each feature. **(C)** ROC curves and AUC values predict survival probabilities at 1, 3, and 5 years. **(D)**. Heatmap of 6 SRlncRNA expression profiles. **(E)** Scatter plot displaying the correlation between glioma patient survival status and risk score. **(F)** A risk score distribution plot depicting the distribution of glioma patients with high and low risk.

To assess the sensitivity and specificity of the riskscore in predicting the survival of glioma patients, the training cohort was categorized into low-risk group and high-risk group. The K-M curve results indicated that patients in the high-risk group had a significantly lower survival rate than those in the low-risk group (*p* < 0.001) (Figure 2A). The area under the ROC curve of the risk score (AUC) was 0.946, which was higher than the AUC values of other clinical parameters (Figure 2B). The ROC curves also gave AUC values of 0.904, 0.947, and 0.897 for overall survival (OS) at 1, 3, and 5 years, respectively (Figure 2C).

**FIGURE 3:**
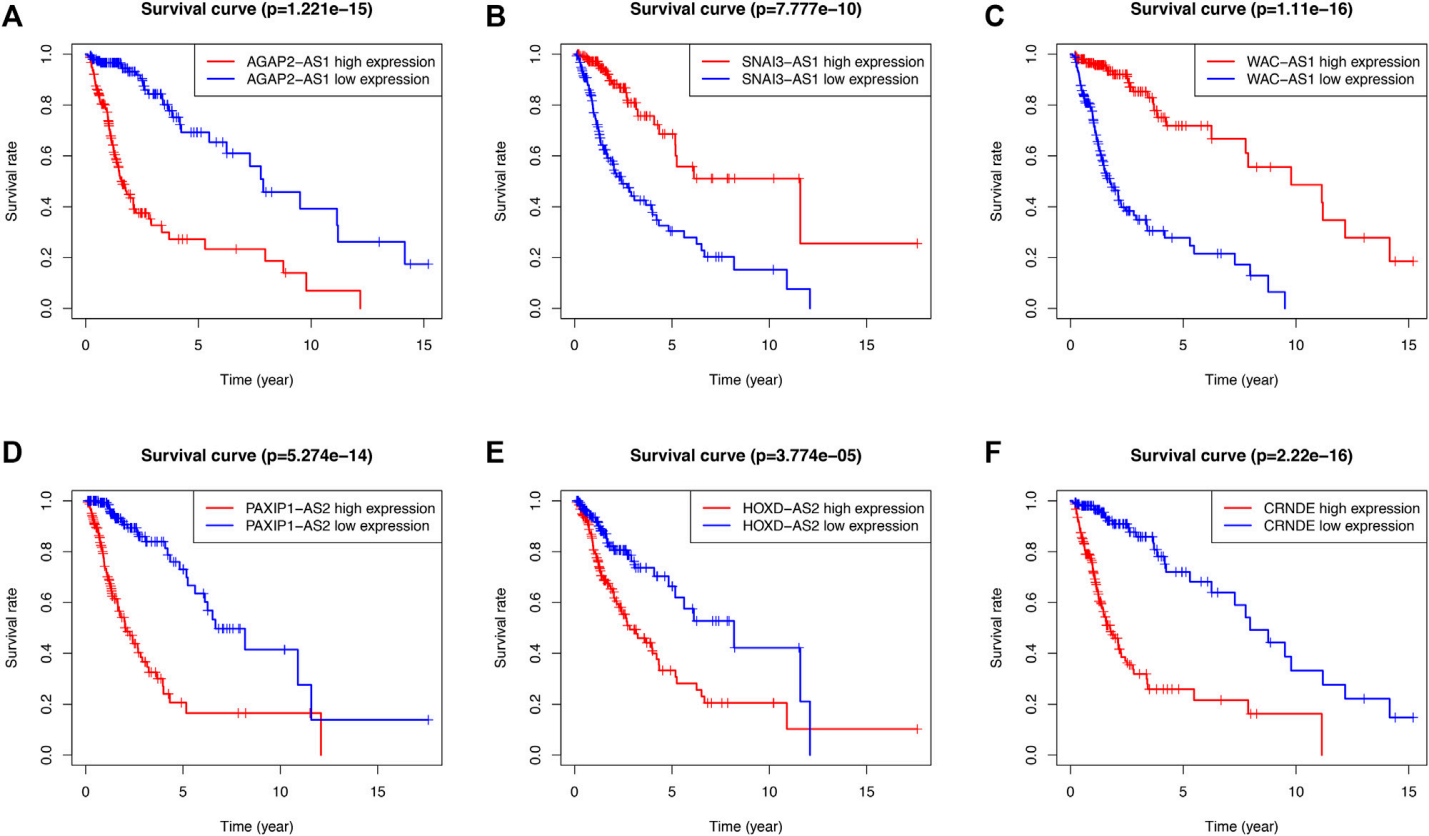
The prognostic signature of SRlncRNA K-M curves in the training cohort. **(A–F)** K-M survival curves for CRNDE, AGAP2-AS1, HOXD-AS2, and PAXIP1-AS2 showed that the high expression group had worse OS, whereas the K-M curves for SNAI3-AS1 and WAC-AS1 showed that the high expression group had better OS.

According to the heatmap, the expression of the 6 SRlncRNAs differed significantly between the high-risk group and the low-risk group (Figure 2D). Patients with glioma who had high risk scores had significantly worse survival rates than those who had low risk scores, as shown in a scatter plot (Figure 2E). Furthermore, the distribution plot of the riskscore corresponded to the patient population categorization (Figure 2F). Further prognostic results of the 6 SRlncRNAs examined by K-M curves revealed that greater expression of CRNDE, AGAP2-AS1, HOXD-AS2, and PAXIP1-AS2 and lower expression of SNAI3-AS1 and WAC-AS1 were substantially linked with shorter OS survival (*p* < 0.0001) (Figures 3A–F). Overall, the results confirmed that the 6 SRlncRNAs formed a highly accurate glioma prognostic risk model.

### 3.2 Validation of the SRlncRNA prognostic signature

**FIGURE 4:**
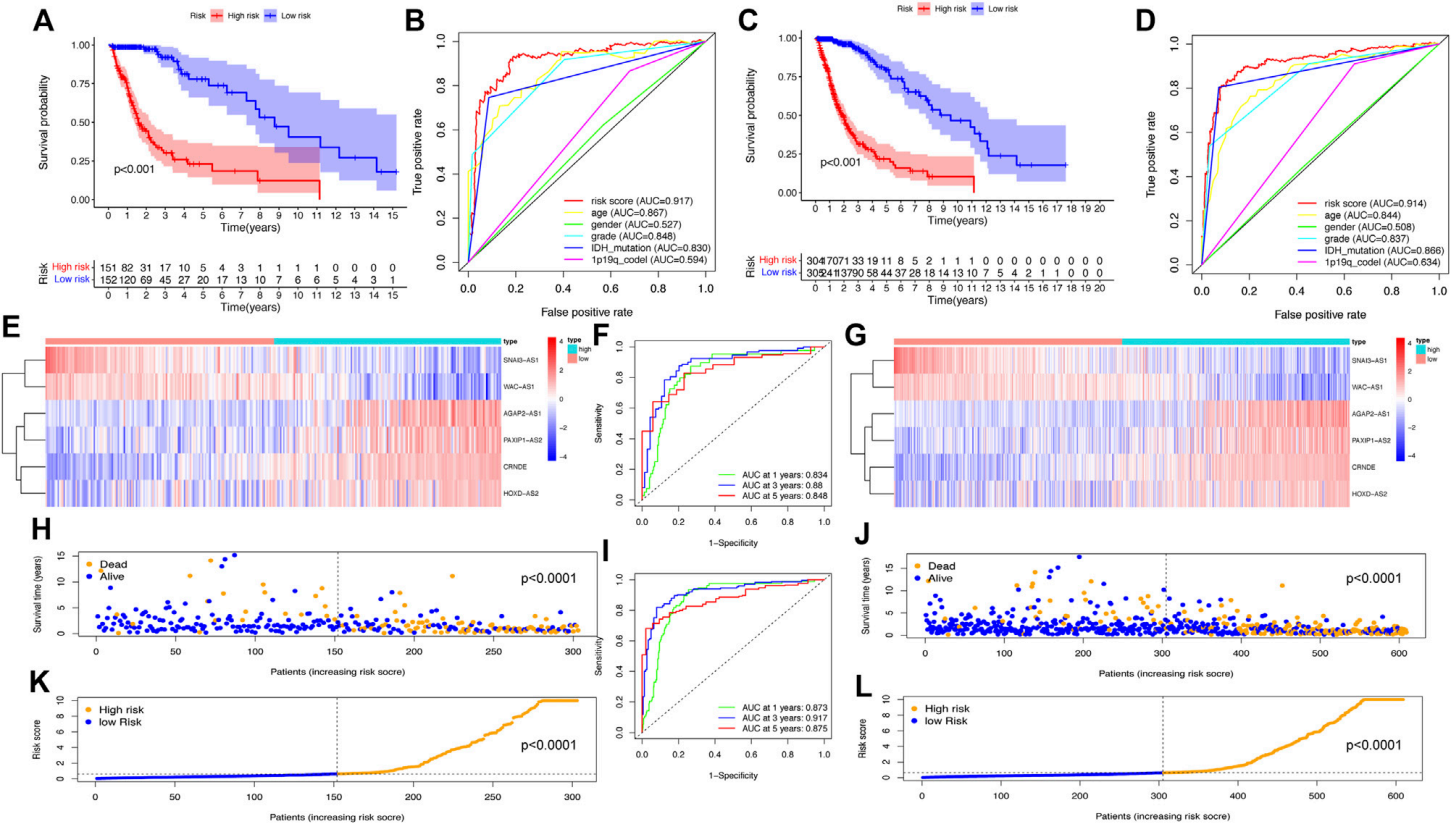
Validation of the SRlncRNA prognostic signature in the testing cohort and the whole cohort. K-M curves show that the higher risk group had worse OS in the testing cohort **(A)** and whole cohort **(C)**. The ROC curves for the prognostic features in the testing cohort **(B)** and whole cohort **(D)** suggest the highest AUC values for the risk score. Heatmap of 9 SRlncRNAs expression profiles show SRlncRNA expression in the high-risk group and the low-risk group in the testing cohort **(E)** and the whole cohort **(G)**. ROC curves at 1, 3 and 5 years in both the testing cohort **(F)** and the whole cohort **(I)** show significant sensitivity. Scatter plots show the correlation between the survival status and risk scores of high- and low-risk glioma patients in the testing cohort **(H)** and the whole cohort **(J)**. Risk distribution plots show the distribution of high-risk and low-risk glioma patients in the testing cohort **(K)** and the whole cohort **(L)**.

To validate the predictive power of the SRlncRNA prognostic signature, the risk scores were calculated for patients in both the testing cohort and the whole cohort, and patients were categorized into the low-risk group and high-risk group based on the median risk score. Analysis of OS by K-M curves for the testing cohort (*p* < 0.001) (Figure 4A) and the whole cohort (*p* < 0.001) (Figure 4C) demonstrated that these results were consistent with the training cohort. The ROC curves for the testing cohort (AUC = 0.917) (Figure 4B) and whole cohort (AUC = 0.914) (Figure 4D) demonstrated the accuracy of the predictions of the SRlncRNA signature for OS in glioma patients, which was verified further by the ROC survival curves and AUC values (Figures 4F, I). The heatmap illustrated the consistent expression profile of the training cohort (Figures 4E, G). Scatter plots indicated worse survival in the high-risk group than in the low-risk group in both the testing cohort and whole cohort, and risk score distribution plots verified a higher risk score in the high-risk group (Figures 4H, K, J, L). Furthermore, the constructed signature was evaluated using the TCGA-LGG, TCGA-GBM cohorts and the CGGA cohorts, the results indicated that the signature was an excellent predictor of survival of glioma patients (Supplementary Figures S1, S2). In conclusion, the SRlncRNA prognostic signature we constructed exhibited good predictive value.

### 3.3 Examine the link between SRlncRNAs and clinicopathological factors

**FIGURE 5:**
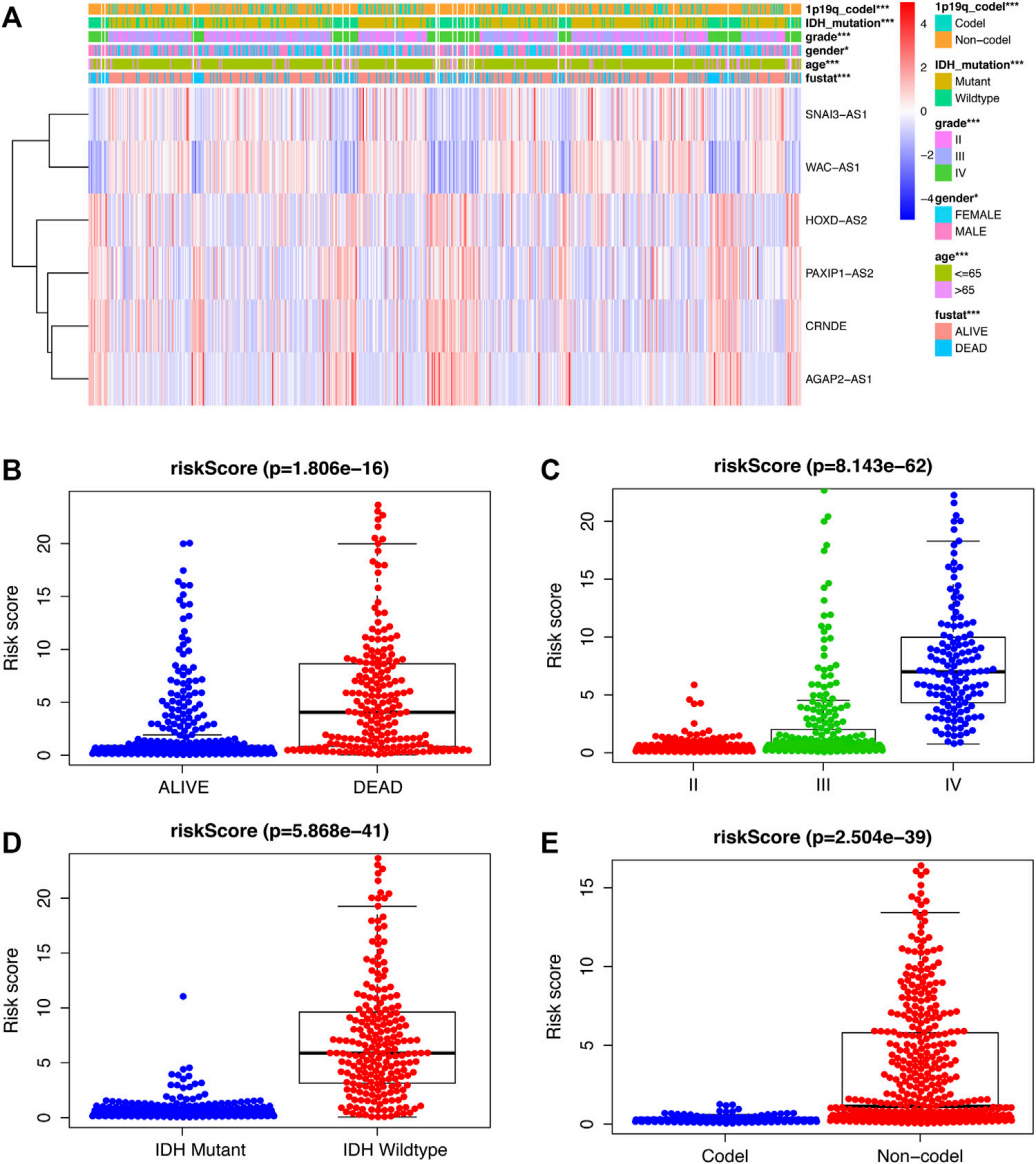
Examination of the link between SRlncRNAs and clinicopathological factors. **(A)** The heatmap suggests that IDH mutation status, 1p19q codeletion status, grade, age, and survival status are significantly different in different groups. **(B,C)** The scatter diagrams indicate that survival status and grade are positive with a higher-risk score. **(D,E)**, The scatter diagrams indicate that IDH mutation status and 1p19q codeletion status are positively correlated with a significantly lower riskscore. *p < 0.05; **p < 0.01; ***p < 0.001; ns, non-significant.

To further assess the role of SRlncRNAs in glioma prognosis, we analyzed the correlation between SRlncRNAs and clinicopathological factors. As shown in Figure 5, the heatmap suggested that IDH mutation status, 1p19q codeletion status, grade, age, and survival status were significantly different in different groups (Figure 5A). The survival status was negatively correlated with a higher risk score (Figure 5B), followed by a positive correlation between glioma grade and a higher risk score (Figure 5C), and there was a lower risk and a better prognosis when an IDH mutation occurred (Figure 5D), as well as a lower risk with 1p19q codeletion (Figure 5E). In conclusion, there is a significant correlation between our constructed glioma risk score and the clinical factors of glioma.

### 3.4 Evaluation of the SRlncRNA prognostic signature for glioma patients

**FIGURE 6:**
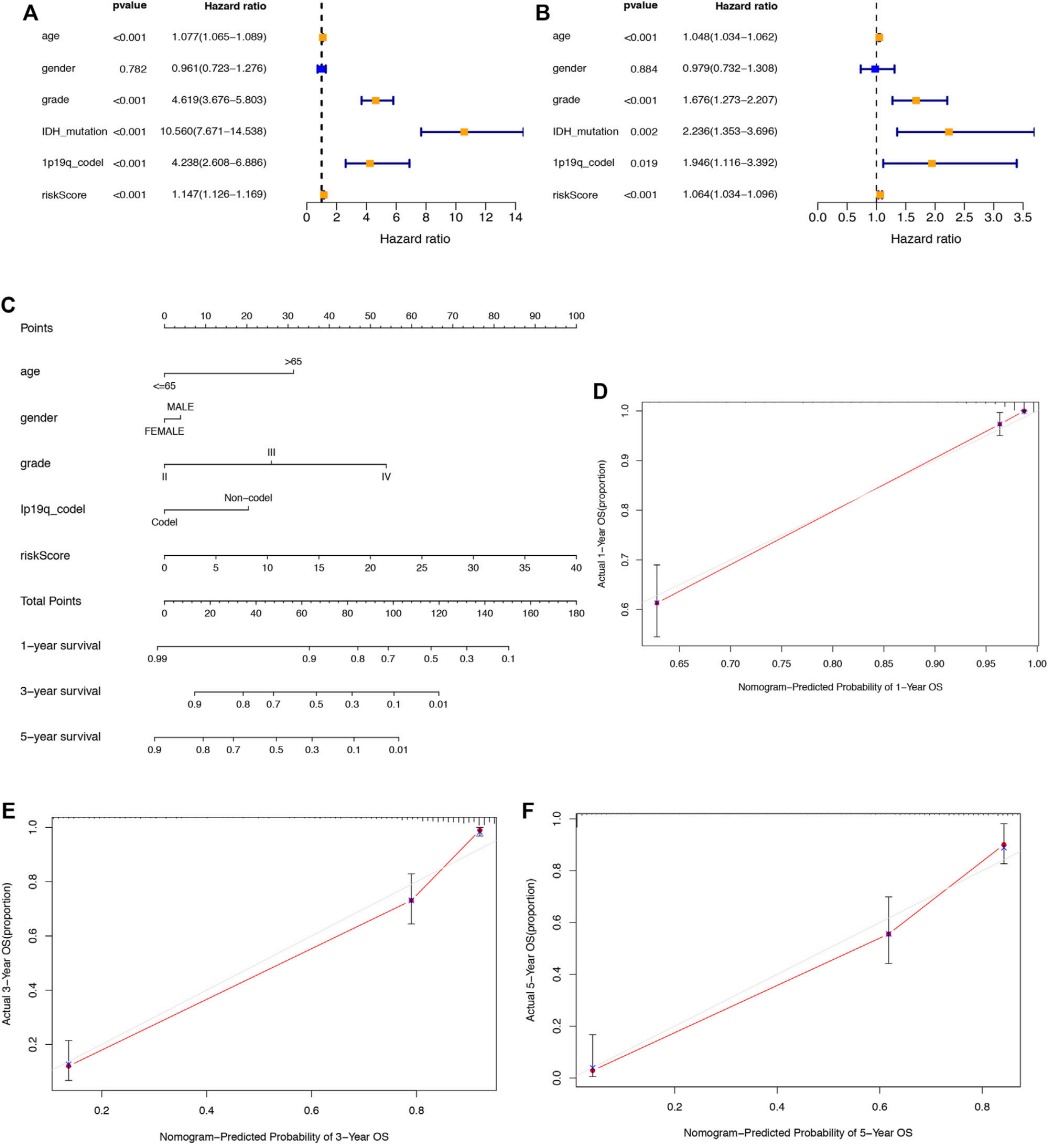
Assessment of the prognostic signature of the SRlncRNAs in glioma. **(A)** The results of univariate Cox regression analysis of the riskscore and clinical factors. **(B)** The results of multivariate Cox regression analysis of the riskscore and clinical factors. **(C)** The nomogram of the risk score and clinical factors. **(D–F)** the calibration curves of the nomogram displaying the concordance between predicted and observed 1-, 3-, and 5-year OS.

Univariate and multivariate Cox regression analyses were used to explore whether the aforementioned 6 SRlncRNA prognostic signature was an independent prognostic signature for glioma. In univariate Cox regression analysis (Figure 6A), the risk score hazard ratio (HR) was 1.147 (95% CI 1.126–1.169) (*p* < 0.001), and in multivariate Cox regression analysis (Figure 6B), it was 1.064 (95% CI 1.034–1.096) (*p* < 0.001). Using age, sex, stage, 1p19q codeletion status, and the risk score, a nomogram plot was constructed to predict 1-year, 3-year, and 5-year survival in glioma patients (Figure 6C). Before the construction of nomogram, Schoenfeld test was performed to examine the quality of factors (Supplementary Figure S4). The nomogram’s prediction capacity was demonstrated by the calibration curve, and the C-index was 0.8547 (Figures 6D–F).

### 3.5 GSEA enrichment

**FIGURE 7:**
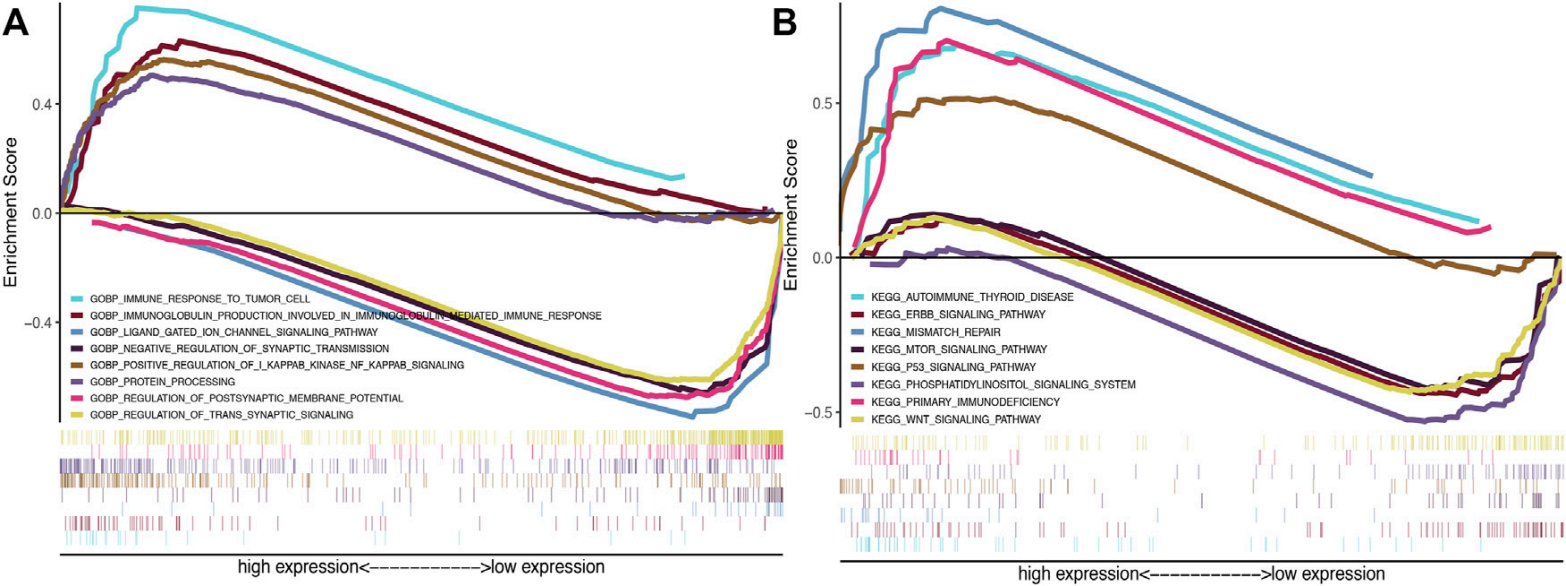
**(A)** Gene Ontology (GO) and **(B)** KEGG analyses of the 6 SRlncRNAs by GSEA.

In the GSEA-GO analysis (Figure 7A), the highly expressed SRlncRNAs were mainly concentrated in the Immune response to tumor cell, immunoglobulin production, synaptic transmission, NF-kB signaling pathway, whereas the SRlncRNAs with low expression were mainly concentrated in ligand gated ion channel signaling pathway, regulation of trans-synaptic signaling. Furthermore, in the GSEA-KEGG analysis (Figure 7B), mismatch repair, immunodeficiency, p53 signaling pathway, autoimmune thyroid disease was enriched in the high expression group, whereas mTOR signaling pathway, WNT signaling pathway, ERBB signaling pathway, phosphatidylinositol signaling system were enriched in the low expression group.

### 3.6 Correlation of SRlncRNAs with the tumor immune microenvironment in glioma

**FIGURE 8:**
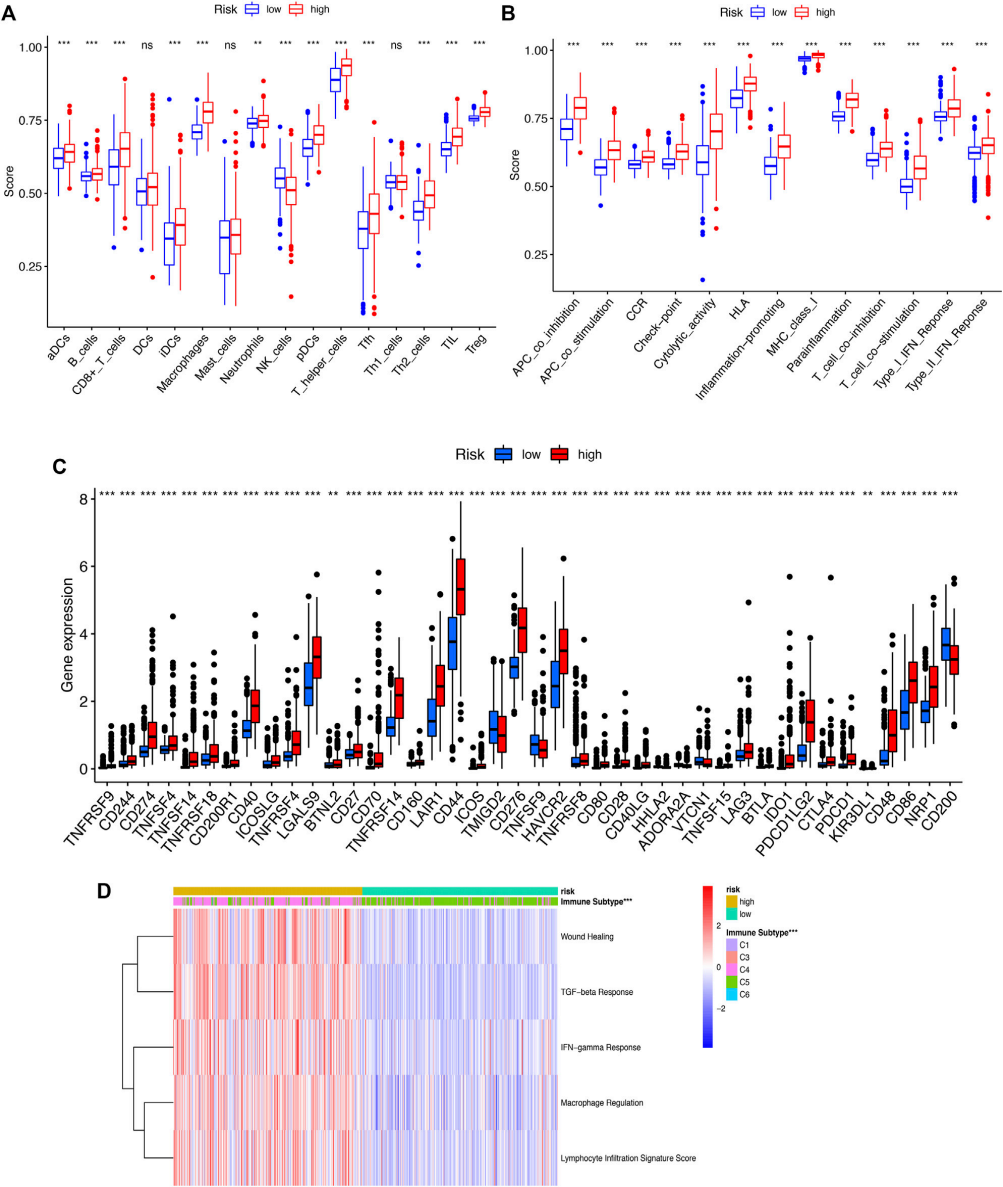
**(A)** ssGSEA algorithm was used to calculate the infiltration levels of 16 immune cells. **(B)**. The correlation between the predictive signature and 13 immun-related functions. **(C)**. The immune checkpoint. **(D)**. The immune subtype (C1-C6). ssGSEA, singlesample gene set enrichment analysis; *p < 0.05; **p < 0.01; ***p < 0.001; ns, non-significant.

To further explore the correlation between riskscore and immune cells, functions and checkpoint, we quantified the enrichment scores of ssGSEA. We found that all items except three immune cells (DCs, NK_cells, Th1_cells) were significantly different (*p* < 0.01, Figure 8A), indicating a significant change in the immunophenotype in the high-risk and low-risk groups. We further explored the expression of immune function and checkpoint-related markers in two groups and found all markers were also significantly different (Figures 8B, C). By assessing the immune subtypes, we discovered that the high-risk group was mostly concentrated in the C4 subtype (Lymphocyte-depleted), was statistically significant, and that the low-risk group was primarily concentrated in the C5 subtype (immunologically quiet) (Figure 8D).

**FIGURE 9:**
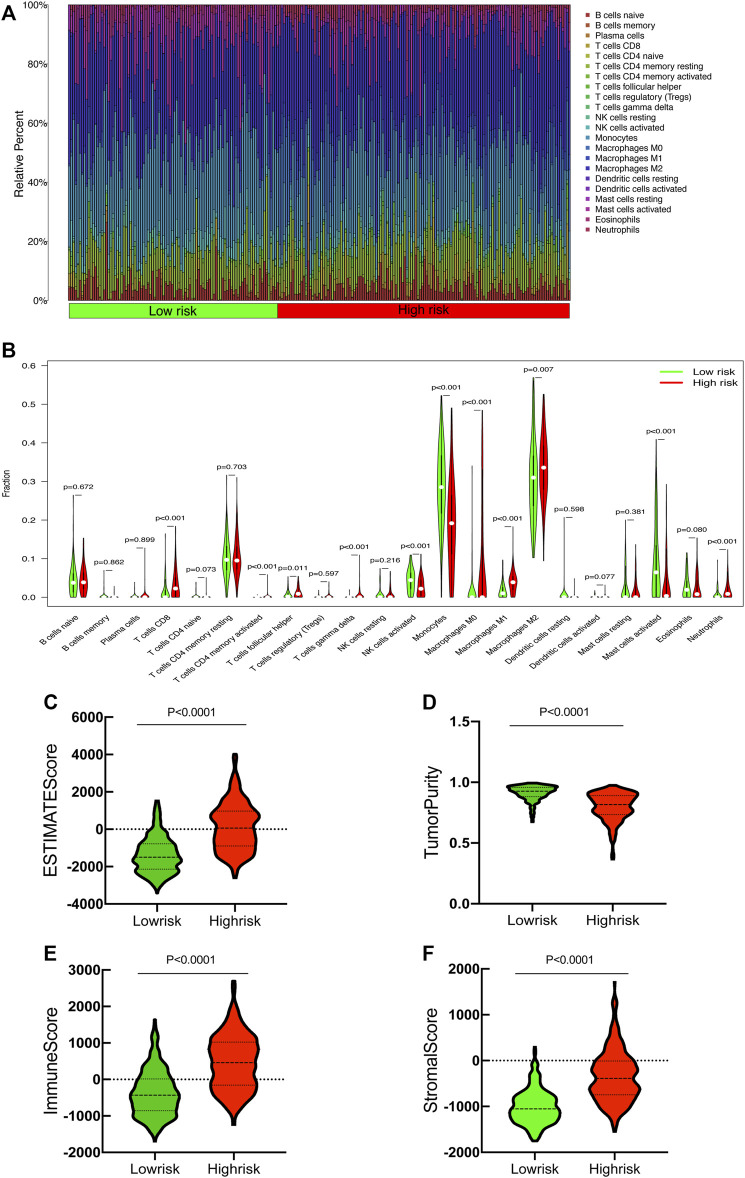
Correlation between tumor-infiltrating immune cells and risk prognosis signature. **(A)** The proportion of 22 immune cell subtypes. **(B)** The correlation between the signature and 13 immune-related functions. **(C–F)** Estimate immune infiltrating score.

The CIBERSORT algorithm was utilized to further evaluate the differences in infiltration of 22 subtypes of immune cells. The relative information proportion of the 22 immune subtypes in the whole cohort and the correlation between the 22 immune subtypes are shown (Figure 9A). Consequently, we investigated the relationship between tumor-infiltrating immune cells and SRlncRNAs. Out of our results, we found that CD8_Tcells, CD4_Tcell memory activated, T-cell follicular helper, T-cell gamma delta, MacrophagesM0-M1-M2 and neutrophils were more abundant in the high-risk group (*p* < 0.05). In contrast, the levels of NK cells activated, Monocytes, and mast cells activated were lower in the high-risk group (*p* < 0.05) (Figure 9B). We also explored the correlation between the SRlncRNA riskscore and tumor-infiltrating immune cells using the ESTIMATE Databases. From the results of ESTIMATE, all items were significant between high-risk and low-risk groups (Figures 9C–F). Based on the aforementioned analysis, we discovered that two groups had a significant and varied immune infiltration pattern, which may have different survival benefits.

### 3.7 Validation of the expression level of SRlncRNAs

**FIGURE 10:**
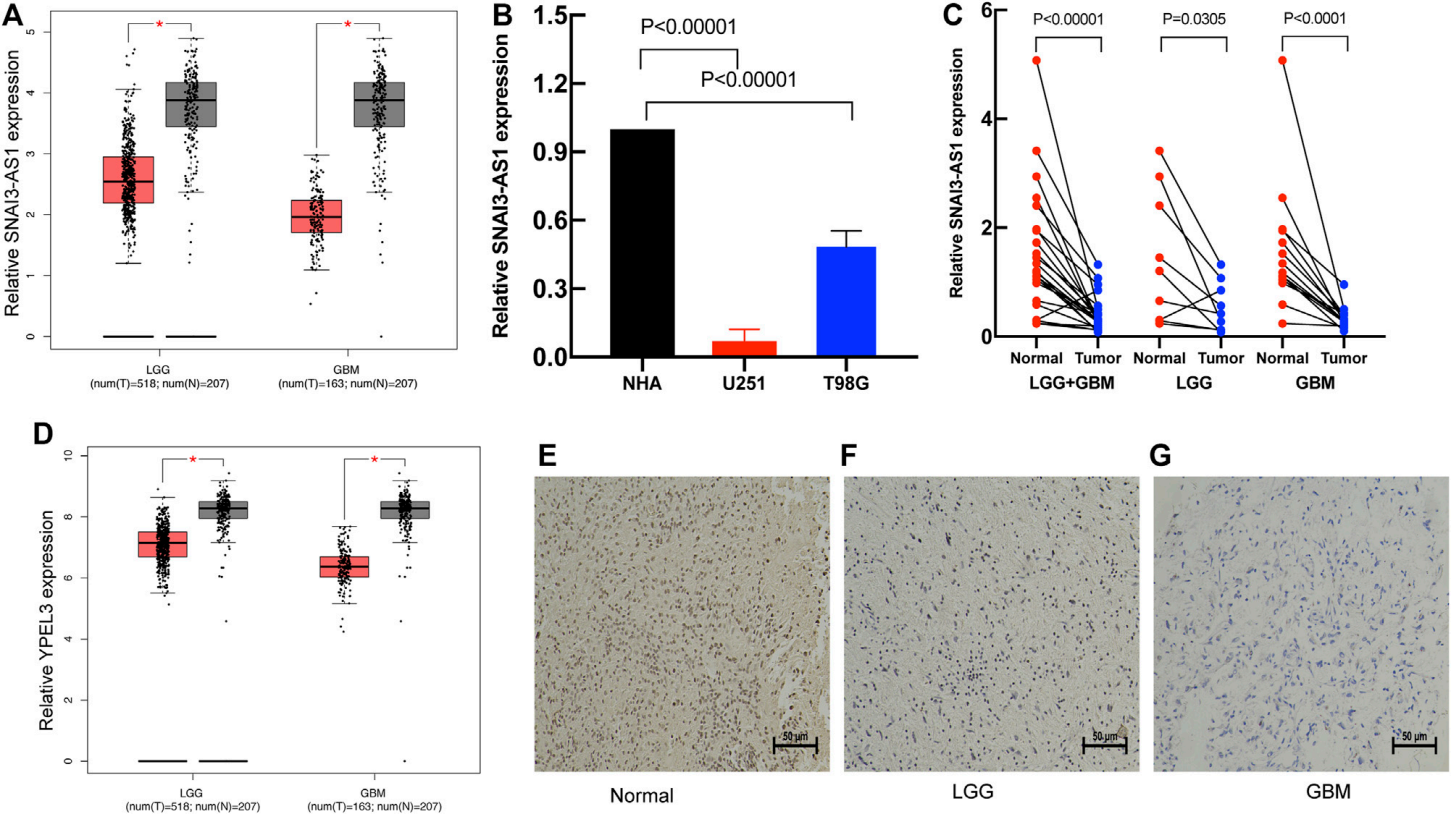
The expression of the SRlncRNA and its target gene. **(A)** The expression of SNAI3-AS1 is shown in GEPIA; **(B)** qRT–PCR was used to detect SNAI3-AS1 expression in cell lines. **(C)** The expression of SNAI3-AS1 was assessed using qRT–PCR in 24 pairs of glioma and matched normal adjacent brain tissue. **(D)** The expression of SNAI3-AS1 target gene (YPEL3). IHC staining for YPEL3 in **(E)** normal brain tissue, **(F)** LGG tissue, and **(G)** GBM tissue.

To further identify the expression levels of SRlncRNAs, we selected SNAI3-AS1 and its target gene for the follow-up analysis. We found that SNAI3-AS1 was significantly under expressed in LGG and GBM by using the Gene Expression Profiling Interactive Analysis (GEPIA) database (Figure 10A). The expression levels of SNAI3-AS1 were confirmed by qRT–PCR in normal human astrocytes and two glioma cell lines, as well as in tumors and adjacent normal tissues from 24 glioma patients (Supplementary Table S4). The results revealed that SNAI3-AS1 was significantly downregulated in glioma cell lines and glioma samples (Figures 10B, C, paired *t*-test). The low expression of its target genes in LGG and GBM was also validated by the GEPIA database (Figure 10D), and the quantity and intensity of the target genes’ immunohistochemical staining in pathological specimens (Figures 10E–G) agreed with the findings above.

## 4 Discussion

Existing risk prediction models for glioma are still insufficient due to its complicated molecular and cellular heterogeneity, and glioma’s high recurrence rate is the leading cause of its mortality (Gusyatiner and Hegi, 2018; Lapointe et al., 2018). To reduce the risk of mortality and recurrence of glioma, a new prognostic model for glioma is urgently needed, and individualized treatment strategies should be developed based on the prognostic model for glioma. Early diagnosis of glioma and timely treatment of recurrences can be achieved if glioma recurrences are detected as soon as possible, aided by close follow-up of high-risk groups.

In this study, SRlncRNAs were screened by calculating the Pearson correlation between lncRNAs and senescence-related genes. Furthermore, LASSO and Cox regression were utilized to obtain the following 6 prognostic SRlncRNAs: SNAI3-AS1, CRNDE, AGAP2-AS1, HOXD-AS2, PAXIP1-AS2, WAC-AS1. The 6 SRlncRNAs were used to establish a risk model for predicting glioma outcomes.

In the current study, all these 6 SRlncRNAs have been previously shown to play significant roles in the pathogenesis and prognosis of cancers. Among them, (Chen et al., 2020), SNAI3-AS1 promoted the proliferation and metastasis of hepatocellular carcinoma by regulating the UPF1/Smad7 signaling pathway (Li et al., 2019b), and SNAI3-AS1 can also promote PEG10-mediated proliferation and metastasis by decoying miR-27a-3p and miR-34a-5p in hepatocellular carcinoma (Li et al., 2020). (Lapointe et al., 2018) CRNDE could regulate the progression and chemoresistance of CRC *via* modulating the expression levels of miR-181a-5p and the activity of Wnt/β-catenin signaling (Han et al., 2017), and CRNDE attenuates chemoresistance in gastric cancer *via* SRSF6-regulated alternative splicing of PICALM (Zhang et al., 2021). (Louis et al., 2021) AGAP2-AS1 could be promising predictive biomarker and therapeutic target for HER-2+ breast cancer patients (Han et al., 2021), and AGAP2-AS1 regulated the proliferation and migration of pancreatic cancer partly through suppressing ANKRD1 and ANGPTL4 (Hui et al., 2019). (Brandner et al., 2022) HOXD-AS2 could promote glioblastoma cell proliferation, migration and invasion by regulating the miR-3681–5p/MALT1 signaling pathway (Zhong and Cai, 2021), (Xu et al., 2022) TENT4A indirectly regulatesRAD18 *via* the tumor suppressor CYLD and *via* PAXIP1-AS2 in endometrial cancer (Swain et al., 2021). (Delgado-Lopez and Corrales-Garcia, 2016) WAC-AS1 can regulate ARPP19 to promote glycolysis and proliferation by sponging miR-320d in hepatocellular carcinoma (Xia et al., 2021). More study is needed to determine exactly how these lncRNAs impact the prognosis of patients with glioblastoma *via* cellular senescence.

Applying the prognostic model to the TCGA dataset, LGGGBM patients were divided into high-risk and low-risk groups. The SRlncRNA signature was validated by the ROC curve, and the AUC value for the risk score-dependent ROC curve was 0.946, which was more sensitive than the AUC of glioma grade. The risk model time dependent AUCs for 1, 3 and 5 years were 0.904, 0.947, and 0.897, respectively, revealing that the SRlncRNA signature performed well in survival prediction.

According to the GSEA results, the differentially expressed genes of the high-risk group and low-risk group were enriched in immune response to tumor cells, mismatch repair, p53 signaling pathway, mTOR signaling pathways, and Wnt signaling pathways. These findings revealed that gene expression differed between the high-risk group and the low-risk group, and SRlincRNA risk scores were linked to the initiation and progression of glioma. In general, the risk score of SRlincRNAs was a fairly accurate predictor of glioma outcomes.

Furthermore, several cancer-promoting or tumor-suppressing lncRNAs, such as NEAT1, H19, NRON, LUCAT1, and HOTAIR, can be found not only in malignant cells but also in tumor-specific immune cells (Willingham et al., 2005; Barnes et al., 2013; Yao et al., 2018; Shin et al., 2019; Agarwal et al., 2020). The immunological functions of cancer-promoting and cancer-suppressing lncRNAs suggest that lncRNAs play a critical role in regulating tumor-immune cell crosstalk during cancer formation and progression (Park et al., 2022). In this study, we explored the correlation between SRlncRNAs and the distribution of tumor-infiltrating immune cells. Apart from CD4 T-cell, the risk scores were shown to be inversely linked with the degree of infiltration of five immune cell types. Our findings indicate that the SRlncRNA risk profile can discriminate between distinct types of tumor-infiltrating immune cells in gliomas. Thus, for the first time, this research explores the involvement of SRlncRNAs and their relationship to the TIME in glioma.

However, we are aware of the limitations of our study. First, we only obtained expression data and clinical data from TCGA for gliomas, and we lacked clinical data for glial-associated methylation. We are still using traditional statistical methods for constructing risk score models of 6 SRlncRNAs, which have been widely used, but more advanced methods are needed, and our current models still need to be validated at the cellular and tissue levels as well as in vivo experiments to ensure our model is more reliable. Overall, we still have many shortcomings to overcome.

## 5 Conclusion

The SRlncRNA signature is accurate and may be used to predict the clinical outcomes and immune microenvironment of patients with glioma, and it may be a useful biomarker and therapeutic target.

## Data Availability

The original contributions presented in the study are included in the article/Supplementary Material, further inquiries can be directed to the corresponding author.
