# Supplementary material for: Identification of a cellular senescence-related-lncRNA (SRlncRNA) signature to predict the overall survival of glioma patients and the tumor immune microenvironment
Source: Front Genet. 2023 Feb 24;14:1096792. doi: 10.3389/fgene.2023.1096792 (PMC9998504; doi:10.3389/fgene.2023.1096792)
Supplement: Supplementary file 1 [file DataSheet1.docx]

**Supplementary Figure S1:** Flowchart of the Study

**
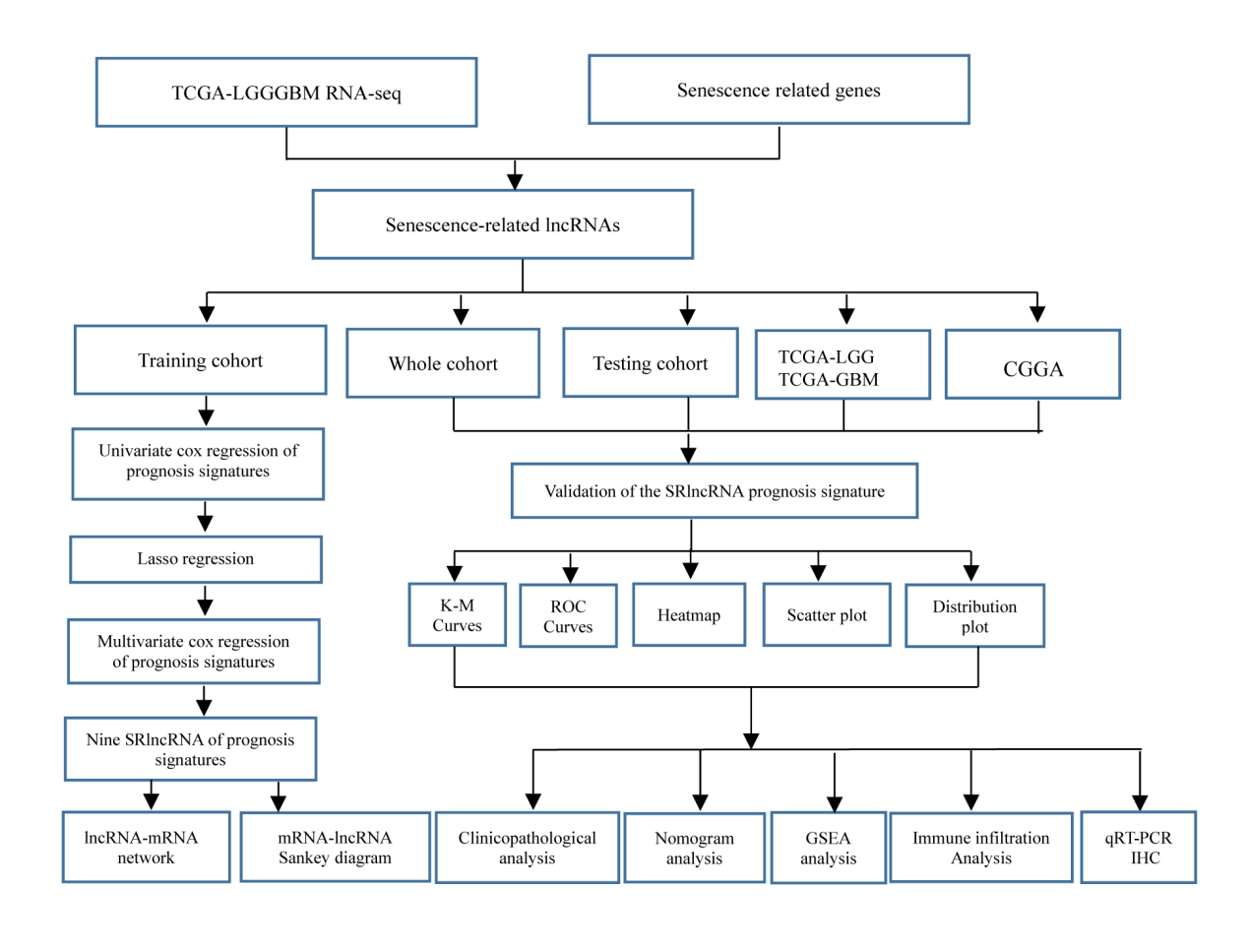
**

**Supplementary Figure S2:** Validation of the SRlncRNA prognostic model in the TCGA-LGG cohort and TCGA-GBM cohort

**
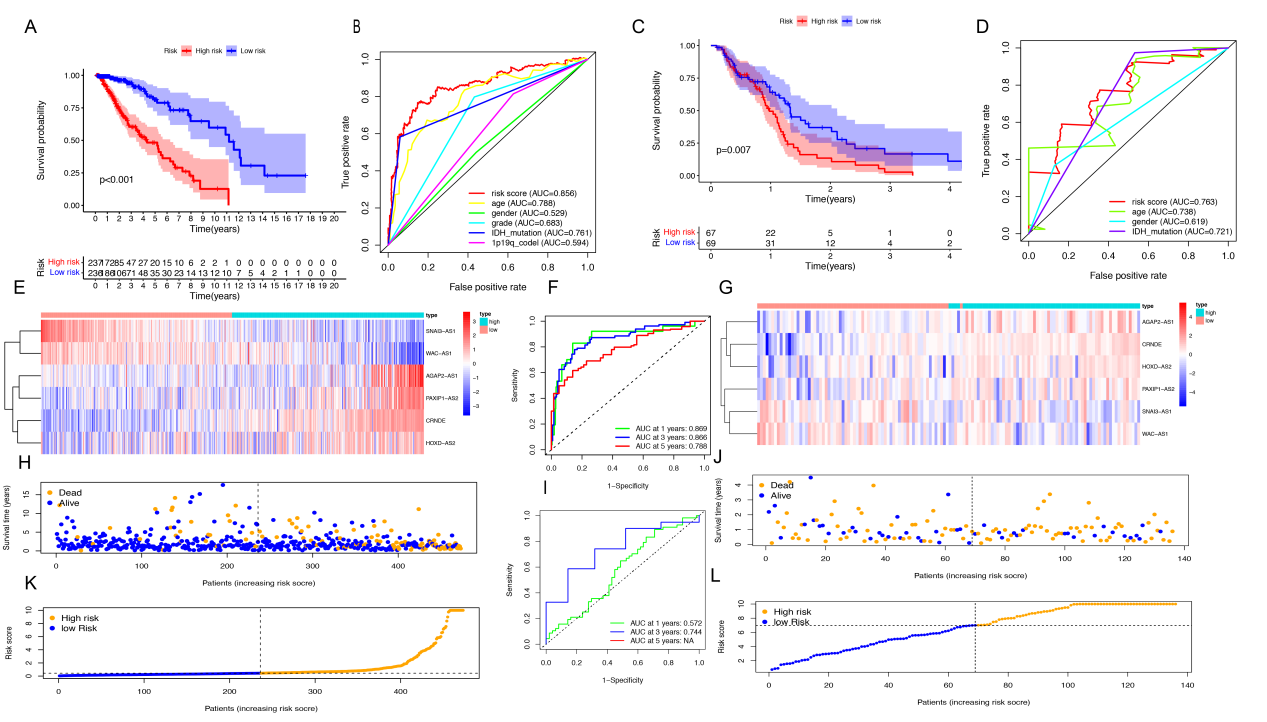
**

**Supplementary Figure S3:** Validation of the SRlncRNA prognostic model in the CGGA cohort

**
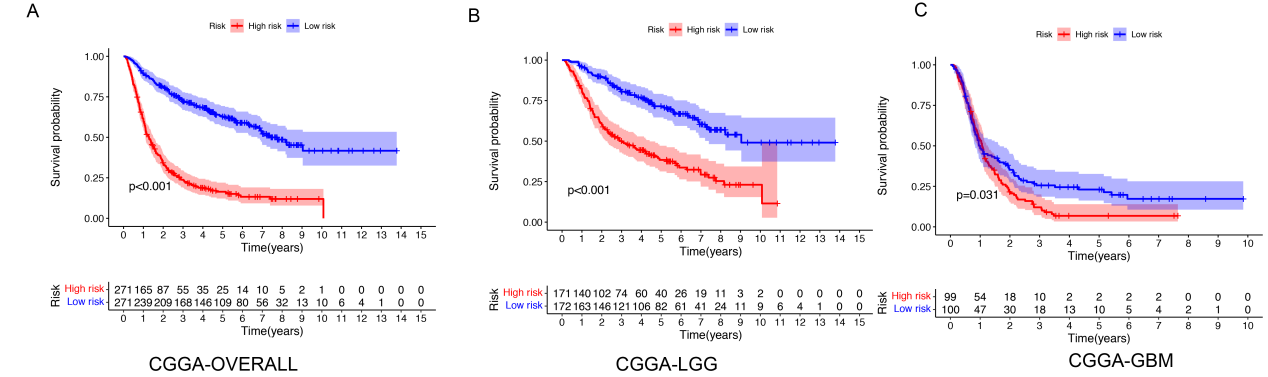
**

**Supplementary Figure S4:** Examine the quality of factors before building nomogram by Schoenfeld test

**
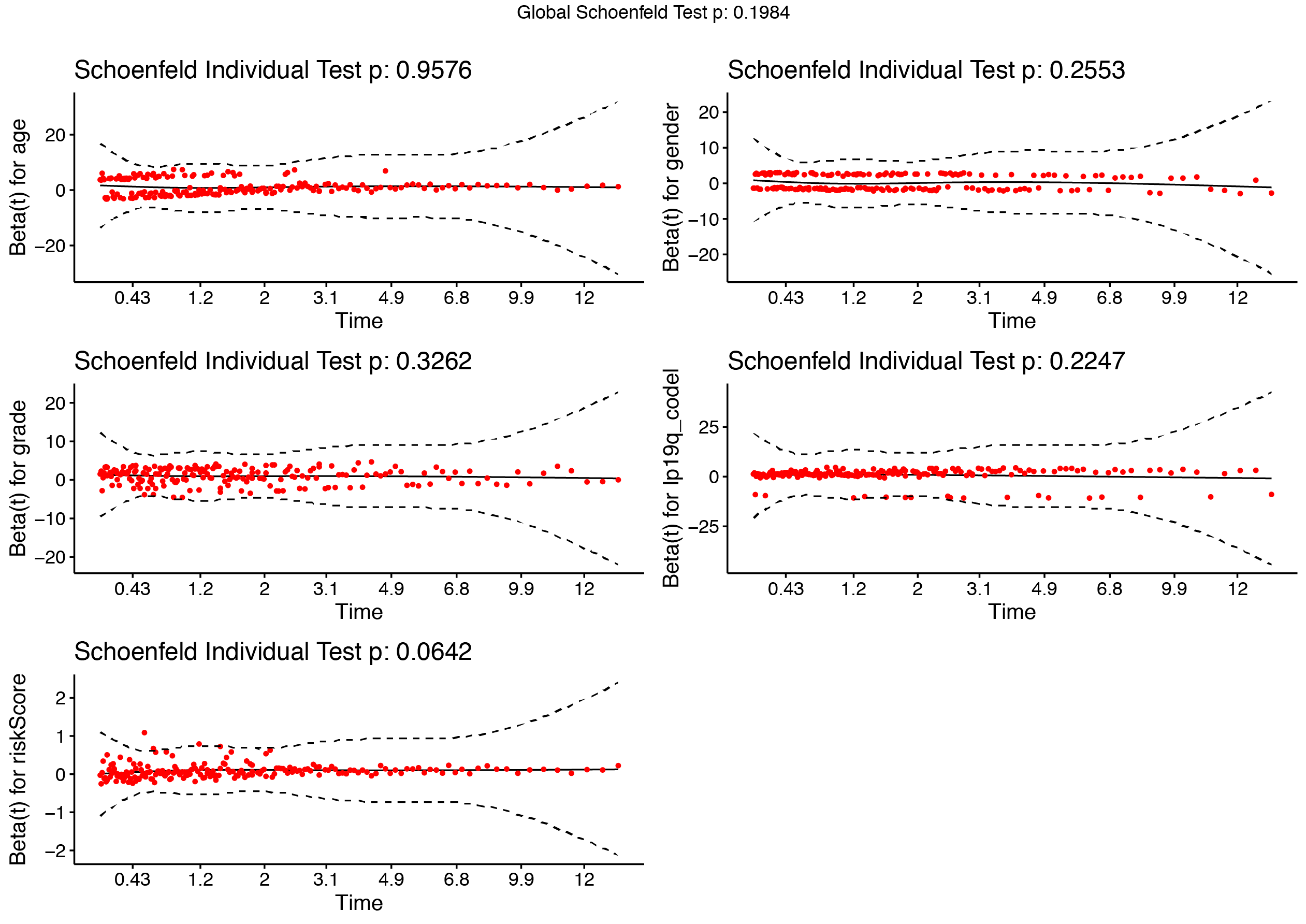
**

**Supplementary Table S1 :** Characteristics of the TCGA-LGG and TCGA-GBM cohorts in this study

| Variable | Group | TCGA-LGG  cohort (n=473) | TCGA-GBM  cohort (n=136) |
| --- | --- | --- | --- |
| Age | ≤65 | 444(93.9%) | 84(61.8%) |
|  | > 65 | 29(6.1%) | 52(38.2%) |
| Gender | Female | 215(45.5%) | 47(34.6%) |
|  | Male | 258(54.5%) | 89(65.4%) |
| Tumor Grade | G2 | 229(48.4%) | 0 |
|  | G3 | 244(51.6%) | 0 |
|  | G4 | 0 | 136 (100%) |
| IDH mutant status | Mutant | 388(82.0%) | 9(6.6%) |
|  | Wildtype | 85(18.0%) | 127(93.4%) |
| 1p19q codeletion status | Noncodel | 317(67%) | 0 |
|  | Codel | 156(33%) | 136(100%) |
| Survival status | Alive | 368(77.8%) | 46(33.8%) |
|  | Dead | 105(22.2%) | 90(66.2%) |
| Survival time (years)（Mean±SD） |  | 2.49±2.54 | 1.04±0.84 |

**Supplementary Table S2:** Characteristics of the CGGA cohort in this study

| Variable | Group | CGGA  Overall cohort (n=542) | CGGA-LGG  cohort (n=343) | CGGA-GBM  cohort (n=199) |
| --- | --- | --- | --- | --- |
| Age | ≤ 65 | 519(95.8%) | 341(99.4%) | 178(89.4%) |
|  | > 65 | 23(4.2%) | 2(0.6%) | 21(10.6%) |
| Gender | Female | 237(43.7%) | 151(44.0%) | 86(43.2%) |
|  | Male | 305(56.3%) | 192(56.0%) | 113(56.8%) |
| Tumor grade | G2 | 141(26.0%) | 141(41.1%) | 0 |
|  | G3 | 202(37.3%) | 202(58.9%) | 0 |
|  | G4 | 199(36.7%) | 0 | 199(100%) |
| IDH mutant status | Mutant | 304(56.1%) | 263(76.7%) | 41(20.6%) |
|  | Wildtype | 238(43.9%) | 80(23.3%) | 158(79.4%) |
| 1p19q codeletion status | Noncodel | 423(78.0%) | 236(68.8%) | 187(94.0%) |
|  | Codel | 119(22.0%) | 107(31.2%) | 12(6.0%) |
| Survival status | Alive | 207(38.2%) | 175(51.0%) | 32(16.1%) |
|  | Dead | 335(61.8%) | 168(48.9%) | 167(83.9%) |
| Survivaltime (years)（Mean±SD） |  | 3.22±2.73 | 4.16±2.79 | 1.61±1.67 |

**Supplementary Table S3 :** LncRNA univariate Cox regression analyses of overall survival in glioma patients

| LncRNA | HR | HR.95 L | HR.95H | pvalue |
| --- | --- | --- | --- | --- |
| LINC01503 | 2.61804 | 2.12093 | 3.23167 | 3.31E-19 |
| AL513534.1 | 0.18129 | 0.11868 | 0.27694 | 2.81E-15 |
| BAIAP2-DT | 0.38292 | 0.31121 | 0.47116 | 1.15E-19 |
| AC092718.4 | 2.66995 | 2.15083 | 3.31435 | 5.45E-19 |
| SNAI3-AS1 | 0.09811 | 0.04791 | 0.20090 | 2.17E-10 |
| AC022007.1 | 4.65732 | 2.94827 | 7.35707 | 4.26E-11 |
| AC025857.2 | 2.62756 | 1.96585 | 3.51201 | 6.75E-11 |
| AC099850.3 | 2.37518 | 1.93253 | 2.91922 | 2.02E-16 |
| CRNDE | 2.28734 | 1.93542 | 2.70325 | 2.82E-22 |
| CYTOR | 2.29242 | 2.00158 | 2.62551 | 4.25E-33 |
| AL355574.1 | 0.28127 | 0.19171 | 0.41268 | 8.85E-11 |
| PLBD1-AS1 | 3.47631 | 2.68367 | 4.50305 | 3.85E-21 |
| FAM181A-AS1 | 2.83947 | 2.25434 | 3.57646 | 7.71E-19 |
| ADGRA1-AS1 | 0.12086 | 0.07229 | 0.20206 | 7.72E-16 |
| AGAP2-AS1 | 1.81487 | 1.64089 | 2.00730 | 4.52E-31 |
| AC048382.5 | 0.11187 | 0.06798 | 0.18408 | 6.73E-18 |
| AL035530.2 | 0.06592 | 0.03796 | 0.11448 | 4.66E-22 |
| AC002454.1 | 2.11353 | 1.78753 | 2.49899 | 2.03E-18 |
| AL133415.1 | 2.57035 | 2.17047 | 3.04390 | 7.24E-28 |
| HOXD-AS2 | 2.28575 | 1.76846 | 2.95434 | 2.70E-10 |
| TMEM220-AS1 | 5.65685 | 4.14169 | 7.72630 | 1.23E-27 |
| AL117332.1 | 7.58683 | 4.95803 | 11.60947 | 9.98E-21 |
| Z95115.1 | 0.20367 | 0.13971 | 0.29692 | 1.30E-16 |
| AC131097.4 | 5.65550 | 4.21624 | 7.58605 | 6.34E-31 |
| PAXIP1-AS2 | 3.42803 | 2.68942 | 4.36949 | 2.50E-23 |
| AL645608.2 | 0.15672 | 0.09089 | 0.27022 | 2.60E-11 |
| WAC-AS1 | 0.19360 | 0.14398 | 0.26033 | 1.66E-27 |
| AC095057.3 | 0.22608 | 0.15424 | 0.33138 | 2.52E-14 |
| AC008669.1 | 0.12561 | 0.08130 | 0.19405 | 8.90E-21 |
| AL590094.1 | 2.72897 | 2.15460 | 3.45645 | 8.35E-17 |
| DGCR9 | 0.24288 | 0.17569 | 0.33578 | 1.09E-17 |

**Supplementary Table S4:** The characteristics of 24 glioma patients

| Variable | Group | Patients(n=24) |
| --- | --- | --- |
| Age | ≤65 | 20(87.0%) |
|  | > 65 | 4(13.0%) |
| Gender | Female | 16(42.9%) |
|  | Male | 8(57.1%) |
| Tumor grade | LGG | 9 |
|  | GBM | 15 |
| Tumor diameter (cm) | <5 | 10 |
|  | ≥5 | 14 |

**Supplementary Table S5: GO Enrichment**

| NAME | NES | NOM p-val | FDR q-val |
| --- | --- | --- | --- |
| GOBP_IMMUNE_RESPONSE_TO_TUMOR_CELL | 1.9795489 | 0.001945525 | 0.091463104 |
| GOBP_IMMUNOGLOBULIN_PRODUCTION_INVOLVED_IN_IMMUNOGLOBULIN_MEDIATED_IMMUNE_RESPONSE | 2.0195735 | 0.005905512 | 0.07584741 |
| GOBP_LIGAND_GATED_ION_CHANNEL_SIGNALING_PATHWAY | -2.0592048 | 0 | 0.052614313 |
| GOBP_NEGATIVE_REGULATION_OF_SYNAPTIC_TRANSMISSION | -2.1306868 | 0 | 0.09030998 |
| GOBP_POSITIVE_REGULATION_OF_I_KAPPAB_KINASE_NF_KAPPAB_SIGNALING | 2.0283415 | 0 | 0.08143862 |
| GOBP_PROTEIN_PROCESSING | 2.1092238 | 0 | 0.21627167 |
| GOBP_REGULATION_OF_POSTSYNAPTIC_MEMBRANE_POTENTIAL | -2.101713 | 0 | 0.056436393 |
| GOBP_REGULATION_OF_TRANS_SYNAPTIC_SIGNALING | -2.0470402 | 0.003745318 | 0.04467457 |

**Supplementary Table S6: KEGG Enrichment**

| NAME | NES | NOM p-val | FDR q-val |
| --- | --- | --- | --- |
| KEGG_AUTOIMMUNE_THYROID_DISEASE | 1.890556 | 0.007707129 | 0.04057126 |
| KEGG_ERBB_SIGNALING_PATHWAY | -1.6031953 | 0.029239766 | 0.18129055 |
| KEGG_MISMATCH_REPAIR | 1.9011582 | 0.003968254 | 0.06053043 |
| KEGG_MTOR_SIGNALING_PATHWAY | -1.5610522 | 0.030864198 | 0.17602843 |
| KEGG_P53_SIGNALING_PATHWAY | 1.7512683 | 0.006048387 | 0.048184376 |
| KEGG_PHOSPHATIDYLINOSITOL_SIGNALING_SYSTEM | -1.6868148 | 0.027613413 | 0.21946733 |
| KEGG_PRIMARY_IMMUNODEFICIENCY | 1.8248291 | 0.009633912 | 0.04775406 |
| KEGG_WNT_SIGNALING_PATHWAY | -1.672065 | 0.021400778 | 0.20567413 |
